# Supplementary material for: Fabrication of Diatomite/Silicalite-1 Composites and Their Property for VOCs Adsorption
Source: Materials (Basel). 2019 Feb 13;12(4):551. doi: 10.3390/ma12040551 (PMC6416632; doi:10.3390/ma12040551)
Supplement: Supplementary file 1 [file materials-12-00551-s001.pdf]

materials

MDPI

Article

Fabrication of Diatomite/Silicalite-1 Composites and their Property for VOCs Adsorption

Yutong Liu and Tao Tian *

Key Laboratory of Groundwater Resources and Environment, Ministry of Education, College of New Energy and Environment, Jilin University, Changchun 130012, China; liuyt841011@163.com
* Correspondence: tiantao@jlu.edu.cn

Received: 18 January 2019; Accepted: 11 February 2019; Published: date

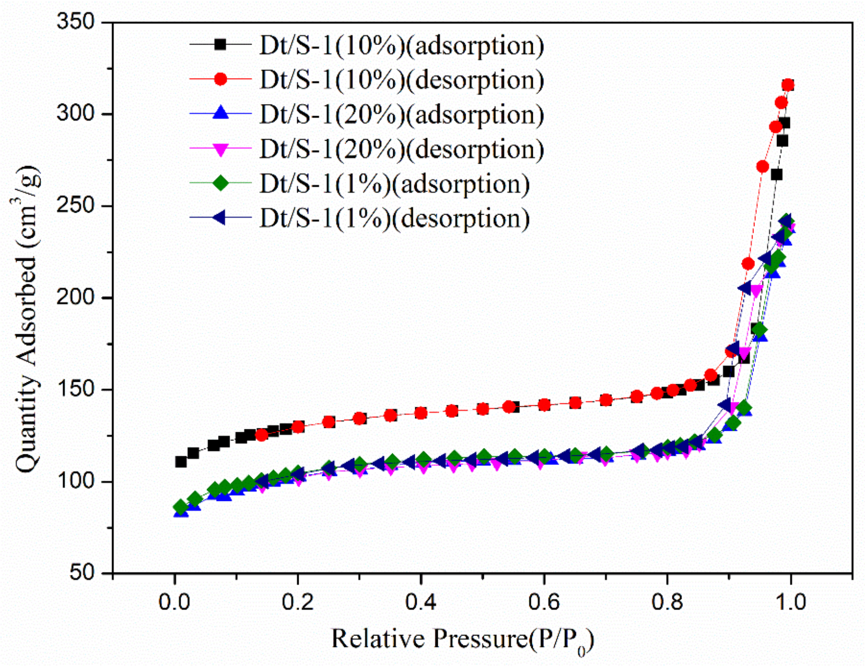

Figure S1. N_2 adsorption–desorption isotherms of Dt/S-1 (1wt%), Dt/S-1 (10 wt%) and Dt/S-1 (20 wt%).

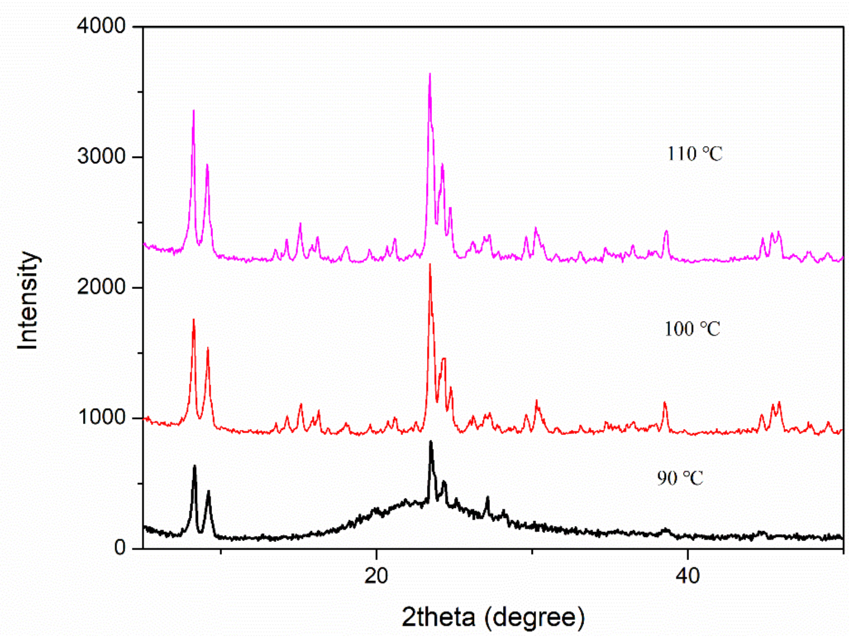

Figure S2. X-ray diffraction (XRD) patterns of the Dt/S-1 (5 wt%) synthesized at different temperatures.

Table S1. Porous parameters of Dt, S-1 and Dt/S-1 (5 wt%).

Sample	Dt/S-1 (1 wt%)	Dt/S-1 (10 wt%)	Dt/S-1 (20 wt%)
S_{BET} (m^2/g)	319.2	402.3	336.8
$V_{micropore}$ (cm^3/g)	0.082	0.133	0.143
V_{total} (cm^3/g)	0.206	0.355	0.216
$W_{zeolite}$ (%)	45.3	75.3	81.2
